# 1462. Validating a claims-based algorithm for Lyme Disease in Massachusetts

**DOI:** 10.1093/ofid/ofac492.1289

**Published:** 2022-12-15

**Authors:** Sheryl A Kluberg, Sarah J Willis, Noelle M Cocoros, Susan R Forrow, Emma R Hoffman, Robert M Jin, Aaron M Mendelsohn, Young Hee Nam, Bradford D Gessner, Sarah J Pugh, James H Stark, Cameron T Nutt, Nathan Petrou, Chanu Rhee, Meera Sury, John Aucott

**Affiliations:** Harvard Medical School & Harvard Pilgrim Health Care Institute, Boston, Massachusetts; Pfizer, Inc., New York, New York; Harvard Pilgrim Health Care Institute, Boston, Massachusetts; Harvard Pilgrim Health Care Institute, Boston, Massachusetts; Harvard Pilgrim Health Care Institute, Boston, Massachusetts; Harvard Pilgrim Health Care Institute, Boston, Massachusetts; Harvard Pilgrim Health Care Institute, Boston, Massachusetts; Harvard Medical School and Harvard Pilgrim Health Care Institute, Boston, Massachusetts; Pfizer Biopharma Group, Collegeville, Pennsylvania; Pfizer, Inc., New York, New York; Pfizer Biopharma Group, Collegeville, Pennsylvania; Brigham and Women's Hospital, Boston, Massachusetts; Brigham and Women's Hospital, Boston, Massachusetts; Brigham and Women's Hospital, Boston, Massachusetts; Brigham and Women's Hospital, Boston, Massachusetts; Johns Hopkins School of Medicine, Baltimore, Maryland

## Abstract

**Background:**

Lyme disease (LD) is the fifth most reported notifiable disease in the US, but the true disease burden remains unknown due to inconsistent reporting. Claims-based algorithms estimate a ≥10-fold higher incidence compared to notifiable-disease surveillance, but these algorithms are unvalidated.

**Methods:**

We evaluated a claims-based LD algorithm based on ICD codes (ICD-9-CM 088.81 or ICD-10-CM A69.2X) and a ≥7-day course of an antibiotic used to treat LD dispensed ±30 days of diagnosis. We applied the LD algorithm to Harvard Pilgrim Health Care (HPHC) claims data for Massachusetts (MA) residents. We sought health records for patients who met the algorithm between Jan 2015 and June 2019 and received care within the Massachusetts General Brigham (MGB) system at diagnosis.

Three clinicians received training on case classification and conducted chart abstractions and adjudications. Cases were classified as confirmed, probable, suspect, or ruled out using 2017 CDC case definitions. To assess inter-rater reliability, the clinicians abstracted and adjudicated the same 20 charts; we computed the mean of kappa for each clinician-pair. We calculated the positive predictive value (PPV) of the algorithm for identifying confirmed, probable, or suspect LD cases, all of which required at least erythema migrans (EM) or clinical diagnosis with confirmatory serology and antibiotics prescription.

**Results:**

We identified 11,823 HPHC members who met the LD algorithm. Of these members, 171 cases occurred within the study period among MGB patients; we obtained 128 (75%) patients’ charts for review. The average weighted kappa statistic of adjudicator agreement was 0.94.

Of the reviewed charts, 103 (80.5%) were adults ≥ 18 years old. 71 patients (55.5%) were clinically diagnosed with LD, among whom 62 (48.4%) presented with EM rash. 24 reviewed cases (18.8%) had laboratory-confirmed LD. LD was ruled out for 8 cases.

The overall algorithm PPV was 93.8% (95% CI 89.6-97.9%). Limited to confirmed and probable cases only, the PPV was 66.4% (95% CI 57.5-74.5%).

**Conclusion:**

A claims-based algorithm combining diagnosis codes and antibiotic prescriptions identified LD cases in MA with high PPV. This algorithm could be used to describe the incidence of LD in regions with similar diagnostic, treatment, and coding practices.

**Disclosures:**

**Sheryl A. Kluberg, PhD, SM**, GlaxoSmithKline: Grant/Research Support|Pfizer, Inc.: Support for the project described in the abstract **Sarah J. Willis, PhD, MPH**, Pfizer: Employment|Pfizer: Conducted Pfizer funded research while employed by Harvard Pilgrim Health Care Institute **Noelle M. Cocoros, DSc, MPH**, Pfizer: PI on study **Bradford D. Gessner, M.D., M.P.H.**, Pfizer Inc.: Employee|Pfizer Inc.: Stocks/Bonds **Sarah J. Pugh, PhD, MPH**, Pfizer, Inc: Employee|Pfizer, Inc: Stocks/Bonds **James H. Stark, PhD**, Pfizer: Employee|Pfizer: Stocks/Bonds **Chanu Rhee, MD, MPH**, Cytovale: Advisor/Consultant|Pfizer: Advisor/Consultant|UpToDate, Inc.: Royalties.

